# Shooting area of infrared camera traps affects recorded taxonomic richness and abundance of ground‐dwelling invertebrates

**DOI:** 10.1002/ece3.11357

**Published:** 2024-04-30

**Authors:** Meixiang Gao, Jiahuan Sun, Yige Jiang, Ye Zheng, Tingyu Lu, Jinwen Liu

**Affiliations:** ^1^ Department of Geography and Spatial Information Techniques Ningbo University Ningbo China; ^2^ Donghai Academy, Ningbo University Ningbo China; ^3^ Shenyang University of Chemical Technology Shenyang China; ^4^ Faculty of Electrical Engineering and Computer Science Ningbo University Ningbo China; ^5^ College of Geography and Environmental Science Hainan Normal University Haikou China; ^6^ Institute of Plant Protection Jilin Academy of Agricultural Sciences Changchun China

**Keywords:** Diplopoda, Formicidae, infrared camera trapping, sampling size, subtropical farmland

## Abstract

Ground‐dwelling invertebrates are vital for soil biodiversity and function maintenance. Contemporary biodiversity assessment necessitates novel and automatic monitoring methods because of the threat of sharp reductions in soil biodiversity in farmlands worldwide. Using infrared camera traps (ICTs) is an effective method for assessing richness and abundance of ground‐dwelling invertebrates. However, the influence that the shooting area of ICTs has on the diversity of ground‐dwelling invertebrates has not been strongly considered during survey design. In this study, data from six ICTs with two shooting areas (A1, 38.48 cm^2^; A2, 400 cm^2^) were used to investigate ground‐dwelling invertebrates in a farm in a city on the Eastern Coast of China from 20: 00 on July 31 to 00:00 on September 29, 2022. Over the course of 59 days and 1420 h, invertebrates within 9 taxa, 2447 individuals, and 112,909 ind./m^2^ were observed from 222,912 images. Our results show that ICTs with relatively large shooting areas recorded relatively high taxonomic richness and abundance of total ground‐dwelling invertebrates, relatively high abundance of the dominant taxon, and relatively high daily and hourly abundance of most taxa. The shooting areas of ICTs significantly affected the recorded taxonomic richness and abundance of ground‐dwelling invertebrates throughout the experimental period and at fine temporal resolutions. Overall, these results suggest that the shooting areas of ICTs should be considered when designing experiments, and ICTs with relatively large shooting areas are more favorable for monitoring the diversity of ground‐dwelling invertebrates. This study further provides an automatic tool and high‐quality data for biodiversity monitoring and protection in farmlands.

## Graphical abstract

Our study was conducted to evaluate the effect that the shooting area of each infrared camera trap has on the taxonomic richness and abundance of ground‐dwelling invertebrates in farmlands.

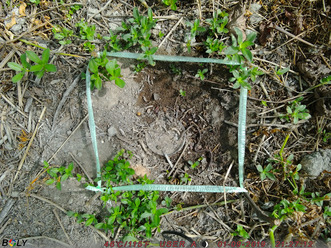

## 1 INTRODUCTION

Ground‐dwelling invertebrates, including pollinators, decomposers, and natural enemies of pests, perform important ecosystem services (Li et al., 2014; Zhukov et al., 2023). Given the serious threat of decreasing soil biodiversity in agricultural farmlands worldwide (Marja et al., 2022; Musters et al., 2022), the use of novel, automatic, and fine temporal resolution methods to monitor and evaluate soil biodiversity is important for the maintenance of the biodiversity of farmlands. Recently, a robust, non‐invasive, and automatic monitoring method, that is, the use of camera traps, has been proposed for monitoring soil biodiversity in farmlands. However, knowledge regarding the utilization of camera traps for monitoring ground‐dwelling invertebrates and their operational standards is limited.

Camera traps can capture images or videos of wildlife without the involvement of humans to trigger the shutter (Meek et al., 2015). Camera traps are proposed and used by researchers and managers to investigate species inventory, monitor species activity and behavior, and monitor biodiversity (Meek et al., 2014). In fact, camera traps have been used extensively to evaluate animal abundance since the 1990s (Karanth & Nichols, 1998). The use of camera traps provides less invasive and potentially more ethical and economical alternatives for evaluating biodiversity. They also facilitate the testing of theories relating to biodiversity maintenance, understanding how ecosystems and their components work, in addition to biodiversity data collection for decision‐making (Nichols et al., 2011). Camera traps with infrared flash, infrared camera traps (ICTs), have been the most widely used in recent field biodiversity monitoring.

The ICTs employ arrays of LEDs that emit infrared light, primarily within the wavelength range of 700–1000 nm. This wavelength is less visible to wildlife than that of incandescent camera traps (Meek et al., 2012). The ICTs also consume less energy than incandescent flashes, and models utilizing them tend to have relatively high trigger speeds (Meek et al., 2012). The ICTs equipped with passive infrared (PIR) sensors can detect heat and motion, and capture photos whenever the PIR sensors detect temperature differences between the ambient environment and the animals' body temperatures. Therefore, the PIR sensors are the most frequently utilized infrared system in ICTs for biodiversity monitoring in the field (Meek et al., 2012). Thus far, the ICTs have been successfully utilized to study the status of species (Can et al., 2011), abundance (Rowcliffe et al., 2011), diversity (Molyneux et al., 2017), habitat use (Liu et al., 2017), activity rhythm (Chen et al., 2019), and functional diversity (Gorczynski et al., 2021) of terrestrial and arboreal vertebrates (Kaizer et al., 2022).

ICTs exhibit certain instabilities resulting from heat differentials that can interfere with PIR effectiveness or when animals are ectothermic, and may not trigger PIR sensors (Corva et al., 2023); however, the developed time‐lapse trigger in ICTs has effectively addressed the issue. The feature enables the setting of intervals between photos, and certain models even permit the capture of both video and still images (Meek et al., 2012, 2014). Ground‐dwelling invertebrates, which are abundant, display diverse diel rhythms (Gao et al., 2023). However, the classical and commonly used methods for collecting ground‐dwelling invertebrates, such as hand sorting (Hashimoto & Mohamed, 2001), pitfall traps (Stašiov et al., 2021), and suction samplers (Frampton et al., 2001), are limited in the distinction of their activities at finer temporal resolutions. Furthermore, such methods have negative impacts on the environment and invertebrates, including the treading habitats, and the mortality of ground‐dwelling invertebrates (Schirmel, Lenze, et al. (2010)), alter the behavior of certain invertebrates, and involve the removal of invertebrates from the target community (Zaller et al., 2015). Such negative effects are particularly evident when conducting high‐frequency surveys. Therefore, employing ICTs with a time‐lapse trigger is an effective and practical approach toward monitoring ground‐dwelling invertebrates in fields with a precise temporal ecological niche across diverse monitoring durations (Burks et al., 2022; Johnson et al., 2020). In recent years, researchers have recommended the use of ICTs to survey ectothermic animals (Corva et al., 2023), including invertebrates (Amorim et al., 2022) in fields.

Owing to the small body size, large number, various speeds of movement abilities, and relatively low body temperatures of ground‐dwelling invertebrates (such as Araneae and Coleoptera), the existing survey designs and standards for monitoring vertebrates and invertebrates using ICTs (Meek et al., 2012, 2014) may not be generally applicable to ground‐dwelling invertebrates. In addition, the selection of appropriate sampling areas for investigative methods is crucial for the evaluation of the biodiversity of ground‐dwelling invertebrates (Boetzl et al., 2018; Jung et al., 2019). Despite being different from the sampling areas of certain classical methods, such as the areas or diameters of pitfall traps, the shooting area utilized by each ICT (i.e., the quantitative capturing zone) must be considered for the standardized monitoring of ground‐dwelling invertebrates. In fact, the standardized performance of experiments not only enables more robust comparisons among related studies but also facilitates study replication (Meek et al., 2014). Ultimately, it advances ground‐dwelling invertebrate studies and facilitates management outcomes. Therefore, during experimental design and implementation, evaluating the impact of ICT shooting areas on the diversity of ground‐dwelling invertebrates is necessary. For example, in pitfall trapping, a standard method for investigating ground‐dwelling invertebrates (Stašiov et al., 2021), large‐diameter pitfall traps more efficiently record more harvestmen, Diplopoda, and Araneae than small‐diameter pitfall traps (Santos et al., 2007; Stašiov et al., 2021). Furthermore, only pitfall traps with large diameters could capture all large‐bodied ant species (Abensperg‐Traun & Steven, 1995). However, the impact of ICT shooting areas on the taxonomic richness, abundance, and density of ground‐dwelling invertebrates has not been comprehensively elucidated.

The choice of shooting areas of camera traps used for vertebrates and invertebrates is related to the body size (Luff, 1975). For large‐bodied vertebrates, the shooting areas of each camera are usually large (Prosekov et al., 2022). For medium‐ and small‐bodied vertebrates, some studies considered the shooting areas of each ICT when setting the experiments. For example, each camera could capture ground‐dwelling mammals and birds entering areas of approximately 2–6 m^2^ (Okabe & Agetsuma, 2007; Samejima et al., 2012). However, these research studies have seldom analyzed the influence of ICT shooting areas on the diversity records of vertebrates (Okabe & Agetsuma, 2007). For ground‐dwelling invertebrates, the shooting areas of each camera are usually smaller than those used for vertebrates. Shooting areas of 2516 cm^2^ (68 × 37 cm) (Zaller et al., 2015) and 300 cm^2^ (Collett & Fisher, 2017) for each digital camera trap were set to investigate ground‐dwelling arthropods in a grassland and forest, respectively. For aboveground invertebrates, the shooting zone of each ICT was usually a focal or a bunch of flowers (Amorim et al., 2022; Naqvi et al., 2022). The main aims of these studies were to reveal the nocturnal activity (Potter et al., 2021), diel patterns (Burks et al., 2022; Johnson et al., 2020), and pollination activity (Amorim et al., 2022) of invertebrates and evaluate the efficiency of different methods of monitoring them (Naqvi et al., 2022). Unfortunately, most of these studies did not evaluate the effect that the shooting area of each ICT may have had on the diversity records of invertebrates. Therefore, the influence of the ICT shooting area on records of ground‐dwelling invertebrates remains unclear.

According to species–area relationships (Lomolino, 2000), as the shooting area expands, the records of taxonomic richness and abundance of ground‐dwelling invertebrates should increase correspondingly. Determining the minimal shooting area for monitoring ground‐dwelling invertebrates and the optimal area where significant disparities are not observed is crucial. However, obtaining this information necessitates comprehensive research. Considering the current scarcity of experiences and examples for monitoring ground‐dwelling invertebrates using ICTs, the authors conducted a preliminary experiment with two distinct shooting areas. One area represented the perimeter and area of pitfall traps (A1, 38.48 cm^2^), which was typically utilized for collecting ground‐dwelling invertebrates. The other represented hand‐sorting squares (A2, 400 cm^2^), where macro‐invertebrates on the soil surface were collected. The aim of the experiment was to determine whether there were disparities between the two commonly applied shooting areas when utilizing ICTs to monitor ground‐dwelling invertebrates, as well as differences between the two areas at varying fine temporal resolutions (day, hour). Because a large ICT shooting area has a large capture zone compared with that of a small ICT shooting area, we hypothesized that ICTs with large shooting areas (A2) would record higher taxonomic richness and abundance of ground‐dwelling invertebrates than ICTs with small shooting areas (A1). The findings of the present study suggest that the shooting area should be considered as a key factor when designing experiments involving ICTs, and could facilitate ICT applications in the biodiversity assessment of ground‐dwelling invertebrates in farmlands.

## 2 MATERIALS AND METHODS

### 2.1 Study site

The study was carried out in Ningbo City (28°51′–30°33′ N, 120°55′–122°16′ E), a coastal city located along the eastern coast of China. The area is situated in the subtropical monsoon climate zone, with an average annual temperature and precipitation of 17.4°C and 1480 mm, respectively (Wang et al., 2022). The temperature maximum and minimum are approximately 39 and −9°C, respectively. The main soil types are red, yellow, and paddy soils (Sun et al., 2020).

The study plots were located in a vineyard of Tiansheng Farm (29°80′ N, 121°40′ E). The area of Tiansheng Farm is approximately 68.7 hm^2^. Vineyards, ryegrass, corn, rice, peanuts, and tobacco plants were being grown in the farmland when the experiment was performed. The vineyard was protected by plastic shed throughout the year.

### 2.2 Setting infrared camera traps and data collection

#### 2.2.1 Setting infrared camera traps

An ICT (BG636‐48M; Boly Media Communications, Santa Clara, CA, USA) was used to photograph ground‐dwelling invertebrates, considering its high‐quality performance and low operational costs. The power supplies for one camera were five alkaline batteries and one solar panel. A 64‐GB capacity secure digital (SD) card was inserted into the camera before setting parameters. The infrared camera was set to capture photos. There are three trigger modes in the infrared camera, that is, PIR trigger, time‐lapse, and a combined PIR/time‐lapse mode. For PIR trigger mode, the camera will activate when motion is detected. For time‐lapse mode, the camera will be active at the set time interval regardless of motion detection. The PIR sensor tends to be less reliable when the temperature differential between the moving objects and ambient temperature is low, for example, <2.7°C (Meek et al., 2012). As ectothermic animals rarely produce temperatures differing from the surrounding environment by 3°C (Hobbs & Brehme, 2017), the ground‐dwelling invertebrates could not trigger the PIR sensor in the present study. Therefore, the time‐lapse mode with a 5‐min interval was used. Additionally, the number of consecutive photographing was set as three, indicating that the infrared camera captured three continuous photos when it was triggered. Therefore, one ICT captured three consecutive photos at 5‐min intervals regardless of motion detection in the present study. Please consult the BG636‐48 M User Manual (https://www.bolymedia.com/Public/upload/PDF/1.71.1BG636E01.pdf) for instructions on how to set up the ICT.

**FIGURE 1:**
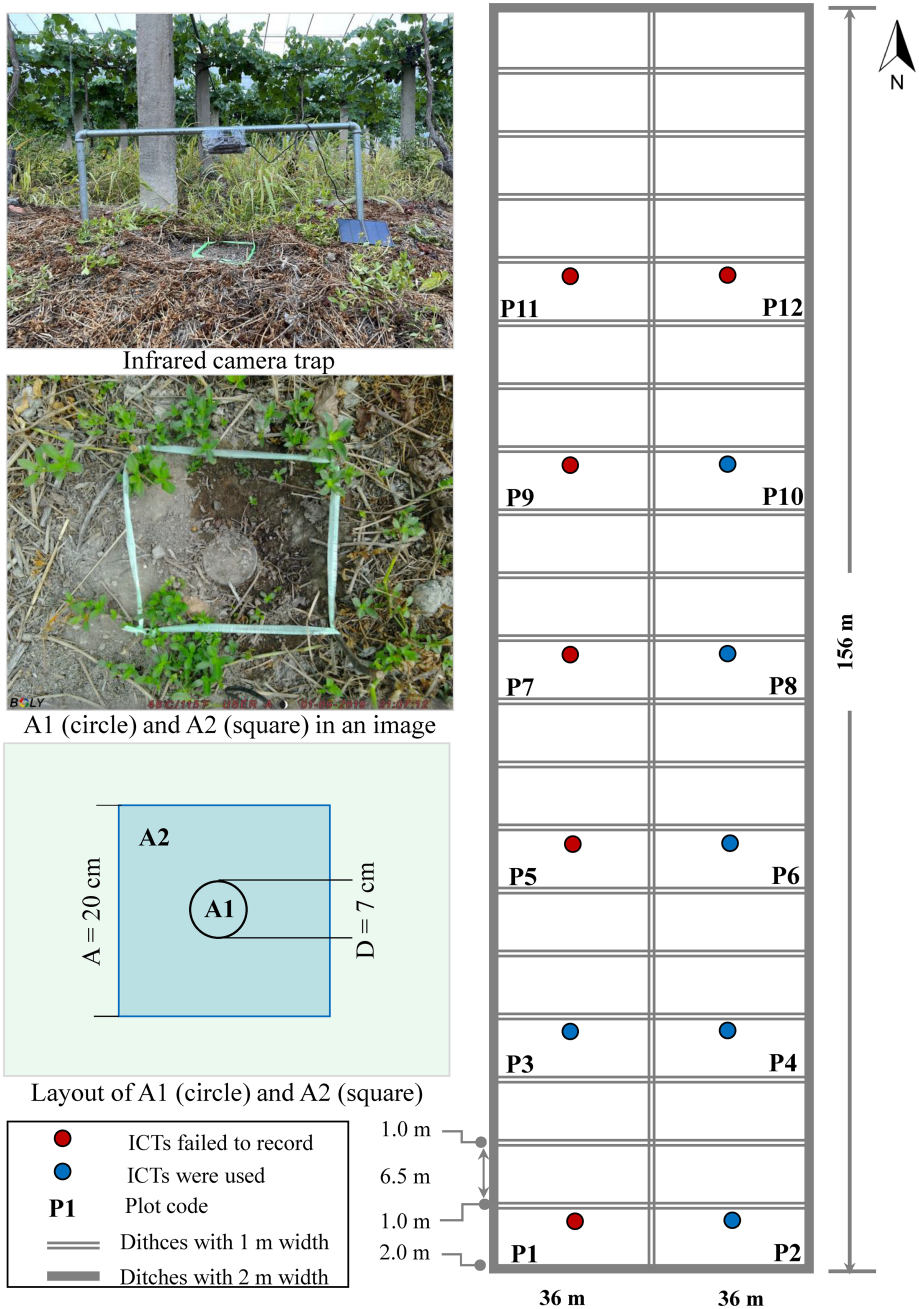
Plot setting.

Twelve plots, each with an area of 234 m^2^, were set up in the farmland and labeled Plots 1–12 (Figure 1). Each plot was separated from adjacent plots with at least one ditch. Each plot contained received water from an irrigation water system and manure and urine from a pig farm, to maintain independence in each ICT plot. One ICT was fixed in the center of each plot. The lens of each camera was set parallel to 40 cm above the soil surface (Figure 1). The cameras set up in the Plots 1–12 labeled as Cameras 1–12, respectively.

The litters directly under each ICT were removed and placed around the ICT before setting the shooting areas, as the invertebrates were difficult to check on the pictures if they were within or under litter. Two shooting areas were set directly under one ICT to make subsequently counting and recognizing clearer and easier, that is, a circular area (*d* = 7.0 cm, area = 38.48 cm^2^, A1) and a square area (20 × 20 cm, area = 400 cm^2^, A2). To minimize the impact of micro‐topography, ground temperature, ground humidity, micro‐soil parameters, grapevines, weeds, and distances to ditch on collected data from A1 and A2 photographed by one ICT, the circular area (A1) was contained within the square area (A2). The center of the circle was located at the center of the square. The lens of the ICT faced the center of the circle and square. As setting up a regular circle in the field is challenging, one pitfall trap (*d* = 7 cm; depth = 10 cm) with a circular mouth was set at the position of the circular area within the square. The pitfall trap was placed into the topsoil with its rim flush to the soil. Thereafter, the pitfall trap was filled with soil to make the surfaces of A1 and A2 flat. The center of the circle rim coincided perfectly with the center of the square. To set the square area, a cardboard with a 20 × 20 cm^2^ area was placed flat on the ground. Four disposable chopsticks were inserted into the soil in the four vertices of the square, and the upper parts of the chopsticks were exposed to the ground by approximately 5 cm. Subsequently, nylon ropes were used to enclose a square above the base of the four chopsticks to make it easier to recognize and count invertebrates in pictures. Ropes were approximately 3 cm parallel and above the ground to prevent them from blocking the activities of the ground‐dwelling invertebrates. To prevent them from loosening and touching the ground following wind and anthropological activity, the ropes were re‐set during each field data collection event. Therefore, invertebrates that entered the circle (A1) and square (A2) areas were photographed by each ICT. The ICTs recorded footage in the plots continuously from 20:00 on July 31 to 00:00 on September 29, 2022—ICTs recorded data for 59 days (1420 h) in total.

#### 2.2.2 Collecting and identifying data

Data were collected on August 8 (July 31–August 8), August 18 (August 8–18), August 25 (August 18–25), September 10 (August 25–September 10), September 18 (September 10–18), and September 29 (September 18–29), 2022. When collecting data in the field, one 64 GB SD with recorded data was taken out from one ICT. After labeling, the SD was placed into an independent plastic bag. Subsequently, one clear 64 GB SD was inserted into the ICT to continue recording. Five batteries were taken out, and five fully charged batteries were inserted into the ICT. In addition, the solar panel was checked to keep it clean and facing the sun. All cameras were ascertained to be working normally, and 12 64‐GB SD and 60 batteries were taken back to the laboratory.

Crops and greenhouses were damaged in the study area following Typhoon 2211 “HINNAMNOR” in early September and Typhoon 2212 “MUIFA” in early mid‐September. Unfortunately, six ICTs (i.e., Camera 1, Camera 5, Camera 7, Camera 9, Camera 11, and Camera 12) were affected considerably by strong winds and storms, and the photos obtained by the 6 ICTs were unavailable or beyond the range of the two monitoring areas (i.e., A1 and A2). Consequently, data from the ICTs could not be used in the subsequent analyses. Therefore, data from the other six ICTs (Camera 2, Camera 3, Camera 4, Camera 6, Camera 8, and Camera 10) unaffected by the weather events were used. In total, 222,912 pictures were recorded.

The ICTs captured vertebrates, such as pigeon, hedgehog, frog, mouse, and snake, and invertebrates in the farmland (Figures S1 and S2). All captured invertebrates were classified into different taxa.

**FIGURE 2:**
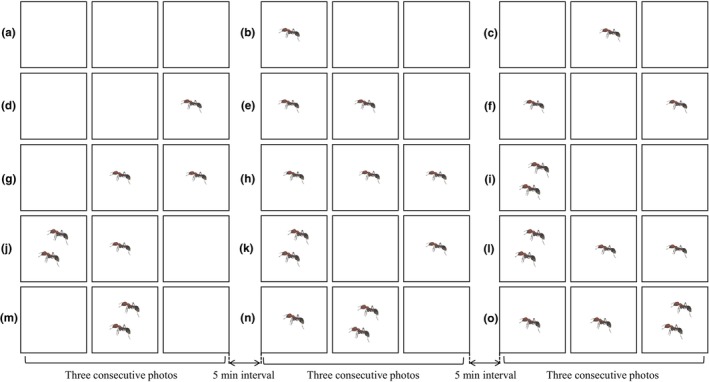
Illustrative examples of identifying and counting the appearance of invertebrates in the pictures. (a) zero Formicidae was photographed; (b–h) one Formicidae was photographed; (i–o) two Formicidae were photographed, at a specific time.

At least three people separately visually identified and classified the invertebrates in each picture. First, the presence of invertebrates in each picture was determined. When an invertebrate appeared in the first, second, or third consecutive photographs, it was deemed to be the same invertebrate at a specific time. When two or more invertebrates appeared in the first, second, or third consecutive photographs, the largest number of invertebrates was counted at a specific time (Figure 2). For example, when one invertebrate appeared in each of the first two consecutive photographs, and two invertebrates appeared in the third photograph, a total of two invertebrates were counted at this specific time (Figure 2o). The photos could only be used in the subsequent process if a consensus was reached that there were invertebrates in the pictures.

Subsequently, the invertebrates in the photos were counted. As different invertebrates exhibited distinct active habits, they were counted based on their activity. According to experience when handling photos with invertebrates, 5 min (i.e., the presented interval) was adequate for the invertebrates with high activity to move away, such as Formicidae, Aranea, and Coleoptera, as the above mentioned invertebrates mostly disappeared from the next three consecutive photos, which were obtained after 5 min. For invertebrates that move relatively slowly, such as Gastropoda (snail) and Oligochaeta (earthworm), some of them still appeared in the next three consecutive photos after 5 min. To address the above, we first decided whether it was the same invertebrate observed in the three preceding consecutive photos obtained 5 min prior. If it was the same, it was counted as a single invertebrate at a specific time point; however, when abundance within an hour was determined, the two photographed invertebrates at two different time points were accounted as one invertebrate. For example, one snail appeared in photos photographed at 23:05, and then the same snail was photographed at 23:10. It was recorded that one snail appeared at the time points of 23:05 and 23:10. Subsequently, they were pooled as one active snail during the 23:00–23:10 period. However, if deciding whether it was the same snail in the photos photographed more than 5 min earlier was deemed challenging, they were defined as different snails.

**FIGURE 3:**
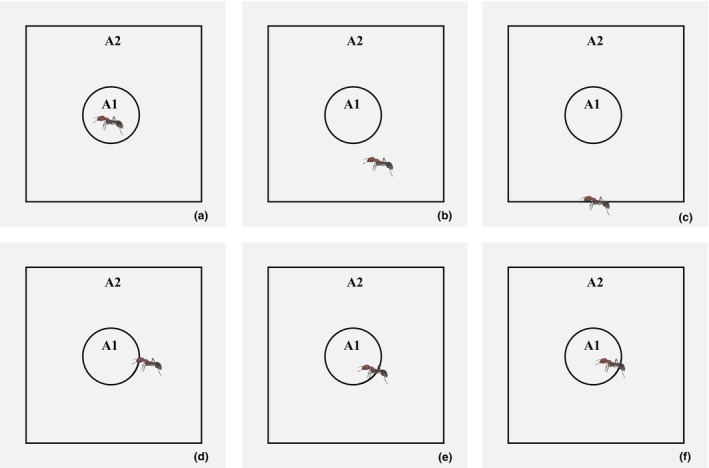
Illustrative examples of counting one invertebrate in one picture which captured by the ICTs. (a, d–f) one Formicidae in A1 and A2 areas, respectively; (b, c) one Formicidae in A2 area.

Subsequently, the invertebrates in each photo were evaluated to belong to A1 or A2. If the whole body of one invertebrate was outside both A1 and A2 areas, it was not counted. If the whole body of one invertebrate was within A1, it belonged to both A1 and A2 areas (Figure 3a). If the whole body of one invertebrate was within A2 but not within A1 areas, it belonged to A2 area (Figure 3b). If a part of the body of one invertebrate was in A2 while the other part was outside A2, it belonged to A2 area (Figure 3c). If a part of the body of one invertebrate was in both the A1 and A2 areas (Figure 3d–f), it belonged to both areas.

Finally, invertebrates were identified in each photo according to published references (Cai et al., 2011; Jiang et al., 2014; Yin et al., 1998).

### 2.3 Data analysis

Taxonomic richness (number of taxa) and abundance (individual number) were calculated to describe the diversity of ground‐dwelling invertebrates captured by each ICT. To test whether differences in observed abundance were due to factors (such as soil micro‐temperature and humidity) other than the shooting areas of the ICTs, abundance was standardized by sampling areas to 1 m^2^, as described in previous studies (Luff, 1975; Work et al., 2002), that is, the activity density (ind./m^2^) of ground‐dwelling invertebrates.

When describing the taxonomic richness, abundance, and activity density of the total ground‐dwelling invertebrates, as well as the abundance and activity density of each taxon in shooting areas A1 and A2, respectively, all invertebrates were pooled throughout the experiment. When describing the daily capture of taxonomic richness, abundance, and activity density of the total ground‐dwelling invertebrates, as well as the daily capture of abundance and density of each taxon in shooting areas A1 and A2, respectively, all invertebrates captured within a 24‐h period were pooled. For instance, the abundance of total ground‐dwelling invertebrates on August 1 was pooled from 00:00 to 24:00 (midnight of the following day). When describing the hourly capture of taxonomic richness, abundance, and density of the total ground‐dwelling invertebrates, as well as the hourly capture of the abundance of each taxon in shooting areas A1 and A2, respectively, all invertebrates captured within a 1‐h period were pooled. For instance, the abundance of total ground‐dwelling invertebrates at 10:00 on August 1 was pooled from 9:00 to 10:00.

A paired t‐test was used to evaluate the differences in richness, abundance, and density of ground‐dwelling invertebrates between A1 and A2 after a logarithmic transformation (log (x + 1)), and *p*‐values <.05 were considered statistically significant. All statistical calculations were carried out in R v 4.2.2 (R Core Team, 2022).

## 3 RESULTS

### 3.1 Total capture of richness, abundance, and density

Nine taxa were captured, namely, Formicidae, Gastropoda (slug), Araneae, Coleoptera, Chilopoda, Diplopoda, Orthoptera, Gastropoda (snail), and Oligochaeta. The taxonomic richness, abundance, and density of total ground‐dwelling invertebrates in A1 were 8, 199, and 51,709 ind./m^2^, respectively; while those indices in A2 were 9, 2448, and 61,200 ind./m^2^, respectively. Formicidae (91.96% [A1] and 78.47% [A2] of all collected invertebrates) were the most abundant in both A1 and A2, followed by Diplopoda (2.51% and 12.99%), Gastropoda (snail) (2.01% and 3.39%), and Araneae (1.01% and 2.94%) (Table 1).

**TABLE 1:** Abundance and density of the total ground‐dwelling invertebrates.

Taxon	Shooting area code	Abundance ± STD	Percentage (%)	Density (ind./m^2^) ± STD
Formicidae	A1	183 ± 53.828	91.96	47,551.6 ± 13,987.039
	A2	1921 ± 417.143	78.47	48,025 ± 10,428.584
Gastropoda (slug)	A1	0	0	0
	A2	7 ± 1.602	0.29	175 ± 40.052
Araneae	A1	2 ± 0.516	1.01	519.69 ± 134.183
	A2	72 ± 6.663	2.94	1800 ± 166.583
Coleoptera (adult)	A1	1 ± 0.408	0.5	259.84 ± 106.081
	A2	36 ± 5.657	1.47	900 ± 141.421
Chilopoda	A1	5 ± 1.329	2.51	1299.22 ± 345.375
	A2	7 ± 1.169	0.29	175 ± 29.226
Diplopoda	A1	5 ± 0.983	2.51	1299.22 ± 255.478
	A2	318 ± 61.381	12.99	7950 ± 1534.519
Orthoptera	A1	0	0	0
	A2	26 ± 5.164	1.06	650 ± 129.099
Gastropoda (snail)	A1	4 ± 1.033	2.01	1039.38 ± 268.367
	A2	83 ± 21.674	3.39	2075 ± 541.853
Oligochaeta (earthworm)	A1	2 ± 0.816	1.01	519.69 ± 212.163
	A2	3 ± 0.837	0.12	75 ± 20.917
Total abundance	A1	199 ± 52.875	100	51,709.12 ± 13,739.297
	A2	2448 ± 481.872	100	61,200 ± 12,046.794
Taxonomic richness	A1	8 ± 0.816		
	A2	9 ± 0.816		

*Note*: Data are the pooled of the total ground‐dwelling invertebrates which monitored during the whole experiment.

**FIGURE 4:**
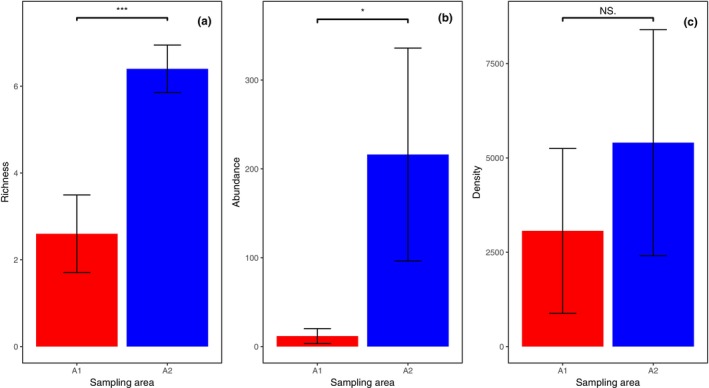
Taxonomic richness (a), abundance (b), and density (c) of the total ground‐dwelling invertebrates in shooting areas A1 and A2 (Mean ± STD). The collected data for the total ground‐dwelling invertebrates were pooled throughout the experiment. * and *** represent *p* < .05 and *p* < .001, respectively. NS., not significant.

The taxonomic richness (Figure 4a; *p* < .001) and abundance (Figure 4b; *p* < .05) of the total ground‐dwelling invertebrates in A2 were significantly higher than those in A1. The density of total ground‐dwelling invertebrates for A1 and A2 did not differ significantly (Figure 4c).

**FIGURE 5:**
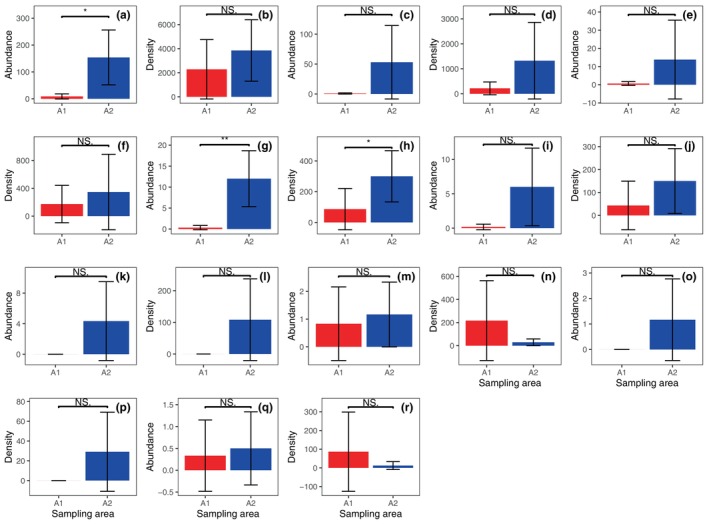
Abundance and density of Formicidae (a, b), Diplopoda (c, d), Gastropoda (snail) (e, f), Araneae (g, h), Coleoptera (i, j), Orthoptera (k, l), Chilopoda (m, n), Gastropoda (slugs) (o, p), and Oligochaeta (q, r) in shooting areas A1 and A2 (Mean ± STD). The collected data for the total ground‐dwelling invertebrates were pooled throughout the experiment. * and ** represent *p* < .05 and *p* < .01, respectively. NS., not significant.

The abundance of Formicidae (Figure 5a; *p* < .05) and the abundance (Figure 5g; *p* < .01) and density (Figure 5h; *p* < .05) of Araneae in A2 were significantly higher than those in A1 throughout the experiment. There were no significant differences in the density of Formicidae (Figure 5b) and the abundance and densities of other taxa between A1 and A2 during the entire experiment (Figure 5c–r).

### 3.2 Daily capture of richness, abundance, and density

**FIGURE 6:**
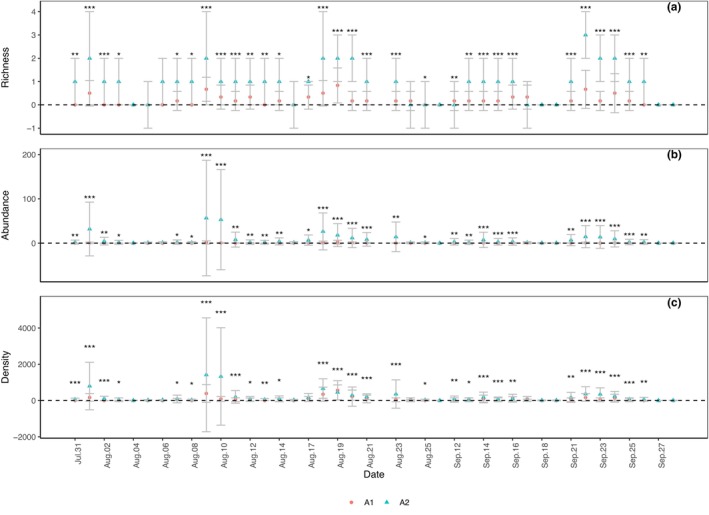
Daily capture of taxonomic richness (a), abundance (b), and density (c) of the total ground‐dwelling invertebrates in shooting areas A1 and A2 (Mean ± STD). Mean and STD represent the average and standard deviation for 24 h each day. *p < .05, **p < .01, and ***p < .001 represent significant differences between A1 and A2 on specific dates.

Daily captures in taxonomic richness (Figure 6a; *p* < .001), abundance (Figure 6b; *p* < .001), and density (Figure 6c; *p* < .001) of total ground‐dwelling invertebrates in A2 were significantly higher than those in A1.

**FIGURE 7:**
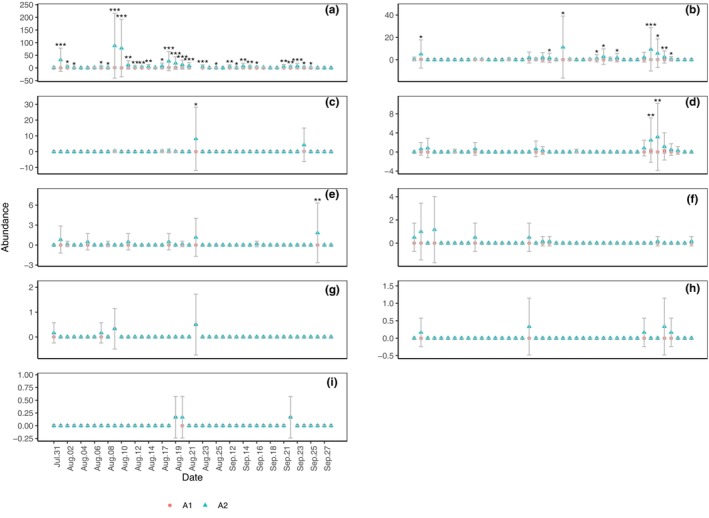
Daily capture of abundance of Formicidae (a), Diplopoda (b), Gastropoda (snail) (c), and Araneae (d), Coleoptera (e), Orthoptera (f), Chilopoda (g), Gastropoda (h), and Oligochaeta (i) in shooting areas A1 and A2 (Mean ± STD). Mean and STD represent the average and standard deviation for 24 h each day. *p < .05, **p < .01, and ***p < .001 represent significant differences between A1 and A2 on specific dates.

The daily abundance of Formicidae (Figure 7a; *p* < .001), Diplopoda (Figure 7b; *p* < .01), Araneae (Figure 7d; *p* < .01), Coleoptera (Figure 7e; *p* < .05), Orthoptera (Figure 7f; *p* < .05), and Gastropoda (slug) (Figure 7h; *p* < .05) in A2 were significantly higher than those in A1, whereas those of Gastropoda (snail) (Figure 7c), Chilopoda (Figure 7g), and Oligochaeta (Figure 7i) were not significantly different. On specific dates, the abundance of certain taxa in A2 were significantly higher than those in A1, specifically for Formicidae (Figure 7a), Diplopoda (Figure 7b), Gastropoda (snail) (Figure 7c), Araneae (Figure 7d), and Coleoptera (Figure 7e).

**FIGURE 8:**
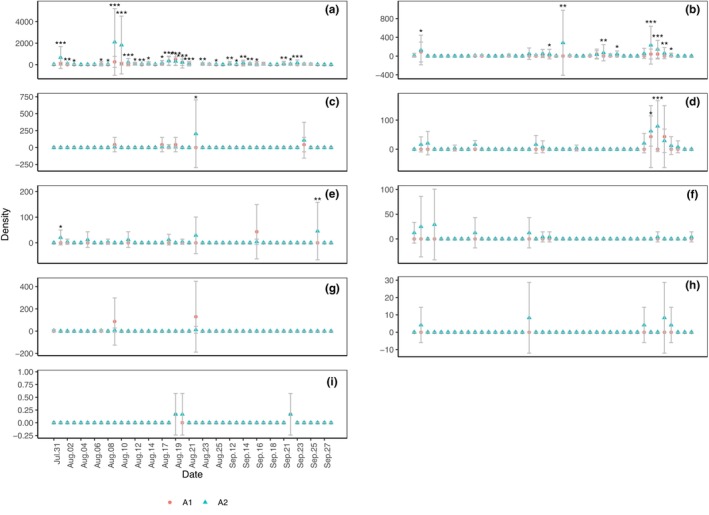
Daily capture of density of Formicidae (a), Diplopoda (b), Gastropoda (snail) (c), Araneae (d), Coleoptera (e), Orthoptera (f), Chilopoda (g), Gastropoda (slugs) (h), and Oligochaeta (i) in shooting areas A1 and A2 (Mean ± STD). Mean and STD represent the average and standard deviation for 24 h each day. *p < .05, **p < .01, and ***p < .001 represent significant differences between A1 and A2 on specific dates.

Daily captures of the density of Formicidae (Figure 8a; *p* < .001), Diplopoda (Figure 8b; *p* < .01), Araneae (Figure 8d; *p* < .05), Orthoptera (Figure 8f; *p* < .05), and Gastropoda (slug) (Figure 8h; *p* < .05) in A2 were significantly higher than those in A1, whereas those of Coleoptera (Figure 8e), Chilopoda (Figure 8g), Gastropoda (snail) (Figure 8c), and Oligochaeta (Figure 8i) were not significantly different. On specific dates, the densities of certain taxa in A2 were significantly higher than those in A1, specifically for Formicidae (Figure 8a), Diplopoda (Figure 8b), Gastropoda (snail) (Figure 8c), Araneae (Figure 8d), and Coleoptera (Figure 8e).

### 3.3 Hourly capture of richness, abundance, and density

**FIGURE 9:**
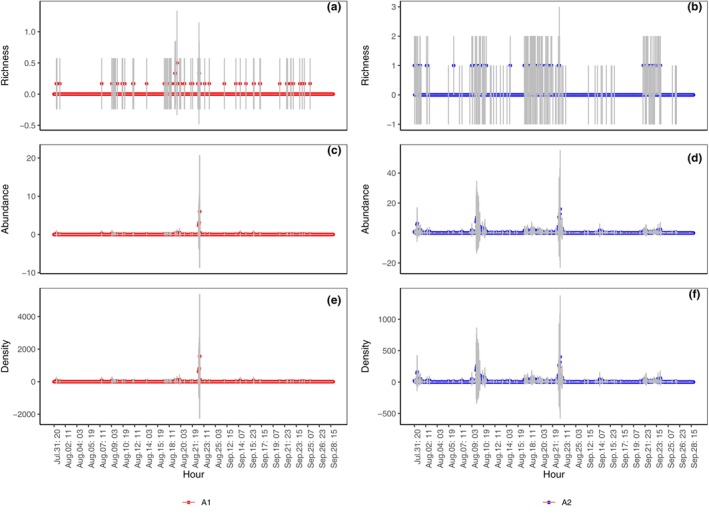
Hourly capture of taxonomic richness (a, b), abundance (c, d), and density (e, f) of the total ground‐dwelling invertebrates in shooting areas A1 and A2 (Mean ± STD), respectively. Mean and STD represent the average and standard deviation, respectively, for 1 h.

Hourly captures in the taxonomic richness (Figure 9a,b; *p* < .001), abundance (Figure 9c,d; *p* < .001), and density (Figure 9e,f; *p* < .001) of the total ground‐dwelling invertebrates in A2 were significantly higher than those in A1.

**FIGURE 10:**
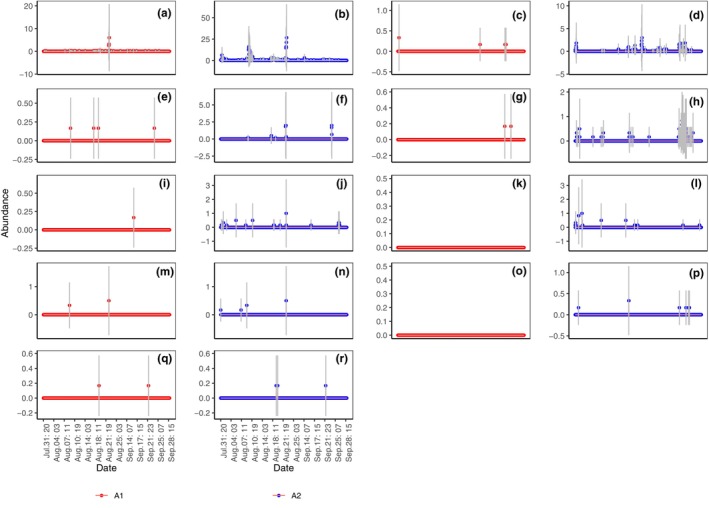
Hourly capture of abundance of Formicidae (a, b), Diplopoda (c, d), Gastropoda (snail) (e, f), Araneae (g, h), Coleoptera (i, j), Orthoptera (k, l), Chilopoda (m, n), Gastropoda (slugs) (o, p), and Oligochaeta (q, r) in shooting areas A1 and A2 (Mean ± STD), respectively. Mean and STD represent the average and standard deviation, respectively, for 1 h.

Hourly captures in the abundance of Formicidae (Figure 10a,b; *p* < .001), Diplopoda (Figure 10c,d; *p* < .001), Gastropoda (snail) (Figure 10e,f; *p* < .01), Araneae (Figure 10g,h; *p* < .001), Coleoptera (Figure 10i,j; *p* < .001), Orthoptera (Figure 10k,l; *p* < .01), and Gastropoda (slug) (Figure 10o,p; *p* < .05) in A2 were significantly higher than those in A1, whereas those of Chilopoda (Figure 10m,n) and Oligochaeta (Figure 10q,r) were not significantly different.

**FIGURE 11:**
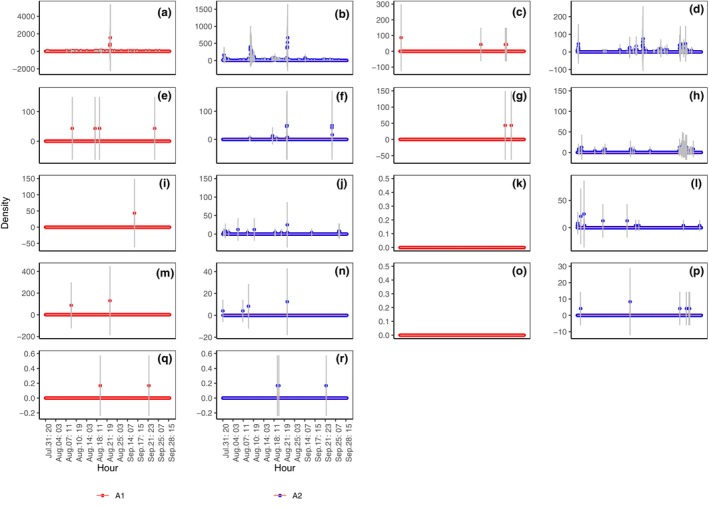
Hourly capture of density of Formicidae (a, b), Diplopoda (c, d), Gastropoda (snail) (e, f), Araneae (g, h), Coleoptera (i, j), Orthoptera (k, l), Chilopoda (m, n), Gastropoda (slugs) (o, p), and Oligochaeta (q, r) in shooting areas A1 and A2 (Mean ± STD), respectively. Mean and STD represent the average and standard deviation, respectively, for 1 h.

Hourly captures in the density of Formicidae (Figure 11a,b; *p* < .001), Diplopoda (Figure 11c,d; *p* < .001), Araneae (Figure 11g,h; *p* < .001), Coleoptera (Figure 11i,j; *p* < .001), Orthoptera (Figure 11k,l; *p* < .001), Gastropoda (snail) (Figure 11e,f; *p* < .05) and Gastropoda (slug) (Figure 11o,p; *p* < .05) in A2 were significantly higher than those in A1, whereas those of Chilopoda (Figure 11m,n) and Oligochaeta (Figure 11q,r) were not significantly different.

## 4 DISCUSSION

### 4.1 Effects of ICTs shooting area on the recorded taxonomic richness and abundance of total ground‐dwelling invertebrates

We found that ICTs with large shooting areas (A2) recorded a higher taxonomic richness and abundance of total ground‐dwelling invertebrates than ICTs with small shooting areas (A1). When considering different taxa, ICTs with large shooting areas recorded a higher abundance of Formicidae and Araneae; however, no significant differences were observed in the abundance of all other taxa between the large (A2) and small (A1) shooting areas. As previous studies on the effects of the shooting area of ICTs on ground‐dwelling invertebrates were scarce, comparatively discussing the results of this study by analyzing similar studies is difficult. Pitfall traps are generally considered the standard method for collecting ground‐dwelling invertebrates. The ecological implications of diameter, size, and area of a pitfall trap are not directly similar to the ecological meaning of the ICT shooting area. For example, pitfall traps physically capture and/or kill the ground‐dwelling invertebrates, whereas ICTs do not physically capture them and potentially recapture their data. However, we compared our results with those from studies that focused on pitfall traps with different diameters or sizes. This comparison was performed to emphasize that shooting area is a key factor influencing the findings of the ICT method and to provide information that could facilitate the effective use of both ICTs and pitfall traps in further research on ground‐dwelling invertebrates.

Luff (1975) stated that pitfall traps with a larger diameter recorded a higher abundance of ground‐dwelling invertebrates, as capture rates were significantly correlated to the circumference of a pitfall trap. In general, large‐sized traps usually collected more ground‐dwelling invertebrates than small‐sized traps (Brennan et al., 1999, 2005). A previous study also showed that the diameter (size) of pitfall traps affects the recorded species richness and abundance of ground‐dwelling invertebrates (Jung et al., 2019). Similarly, the current study showed that the shooting areas of ICTs significantly affected the recorded taxonomic richness and abundance of the total ground‐dwelling invertebrates. ICT records of ground‐dwelling invertebrates in the present study evaluated the relative abundance rather than the absolute density of ground‐dwelling invertebrates (Southwood, 1994). Luff (1975) suggested that if the sizes of pitfall traps were the only factor influencing capture rates, the relative abundance among different sizes of pitfall traps should be indistinguishable when capture rates are standardized. No difference was observed in the abundance of total ground‐dwelling invertebrates once the relative abundance was standardized by sampling areas to 1 m^2^ (i.e., density). These results emphasized that the shooting area of ICTs was an important factor (there may likely be more) that affected the recorded abundance of total ground‐dwelling invertebrates.

Santos et al. (2007) showed that small‐sized and larger pitfall traps both collected the dominant fauna. However, ICTs with small shooting areas recorded the dominant taxon (Formicidae) in significantly lower numbers than ICTs with large shooting areas in the current study. The capture rates of ICTs might depend on the taxonomic activity and density. Formicidae were the most abundant taxon captured by ICTs with both large and small shooting areas. Formicidae are active invertebrates with relatively quick movements compared with those of the other collected invertebrates found in the study area; therefore, there would likely be more opportunities for Formicidae to move across the shooting areas of the ICTs used in this study. This reasoning likely explains why ICTs with large shooting areas recorded a higher abundance of Formicidae compared with that of ICTs with small shooting areas. However, after the abundance of Formicidae was standardized based on 1 m^2^, no significant differences were observed between the density of Formicidae in the two ICT shooting areas, indicating that the difference in abundance of Formicidae observed between A1 and A2 was mainly due to an increase in the ICT shooting area (Work et al., 2002).

Brennan et al. (1999) found that pitfall traps with a large size recorded a higher species richness for Araneae. Work et al. (2002) also demonstrated that larger pitfall traps captured more Araneae of all body sizes. When using ICTs to capture Araneae in the present study, ICTs with larger shooting areas recorded a higher abundance of Araneae than those with small shooting areas. When the abundance was standardized by 1 m^2^, the density of Araneae in the two shooting areas remained significantly different. This differed from the findings of a similar study carried out in a forest, which found no significant difference in the abundance of Araneae among different trap sizes after standardization (Work et al., 2002). When using ICTs to investigate ground‐dwelling Araneae in the present study, apart from shooting area, other potential factors were also important, such as the soil micro‐temperature, soil micro‐humidity between A1 and A2 and Araneae's movement ability.

Both the abundance and occurrence rates measured among the six ICTs for the other taxa, such as Gastropoda (slug), Oligochaeta, and Chilopoda, were low. Certain members of some taxa (Gastropoda [snail] and Oligochaeta) move much slower than those of other taxa. For such taxa, ICTs detected non‐significant differences between abundance in large and small shooting areas for the other taxa. The disproportionate abundance of Formicidae and the other taxa might be an important factor influencing the capturing efficiency of ICTs with different shooting areas. The use of more ICTs or additional methods, such as a combination of ICTs and pitfall traps, to collect rare taxa of ground‐dwelling invertebrates has been suggested (Lindsey & Skinner, 2001; Work et al., 2002).

### 4.2 Effects of ICTs shooting area on the recorded temporal dynamics of the taxonomic richness and abundance of ground‐dwelling invertebrates

ICTs with large shooting areas recorded higher daily and hourly captures of taxonomic richness and abundance of the total ground‐dwelling invertebrates than ICTs with small shooting areas. The significant difference between the abundance of total ground‐dwelling invertebrates for the two different shooting areas remained once the relative abundance was standardized based on 1 m^2^ for daily and hourly captures. These results indicated that the daily and hourly dynamics in the abundance of total ground‐dwelling invertebrates were mainly due to the increased shooting area, whereas important factors (such as micro‐environment) likely also affected their dynamics.

ICTs with large shooting areas recorded higher daily and hourly captures in the abundance of most taxa except those with low abundance, such as Oligochaeta, Gastropoda (slug), and Chilopoda. A significant difference was observed between both the daily and hourly captures of the abundance of most taxa for large and small shooting areas once standardized to sampling areas of 1 m^2^. These results indicated that the shooting areas of ICTs and other unmeasured factors affected the daily and hourly captures in the abundance of most taxa observed in this study significantly.

Although the daily and hourly abundance of invertebrates are important data for biodiversity assessment and protection, related studies are still scarce. Scientists have investigated the daily oviposition and diurnal change in adult behavior of beech caterpillars using camera traps (Kamata & Igarashi, 1995). In a study using ICTs that lasted 560 days and 8230 trap nights, researchers detected hourly, diel, and seasonal dynamics in the abundance of terrestrial leeches in a Malaysian rainforest and found that air humidity rather than host abundance determined leech abundance (Jambari et al., 2022). Over approximately 190 days, the diel, daily, and monthly dynamics of navel orange worm males were reported via the use of ICTs (Burks et al., 2022). Results of these studies showed that ICTs are a useful tool for investigating fine temporal dynamics of invertebrates. The present study further evaluated the effect of shooting areas of ICTs on the daily and hourly captures of invertebrates. However, a notable spike was identified in abundance around August 22, especially for Diplopoda, Gastropoda, Coleoptera, and Chilopoda. During this period, the study areas experienced extreme high temperature and drought events in 2022 (Ningbo Climate Center, 2022). Irrigation activities by managers and random invertebrate processes could have been responsible. However, our results revealed that using ICTs could provide a useful approach for investigating the temporal dynamics of ground‐dwelling invertebrates in different shooting areas at fine temporal resolutions.

When evaluating the daily and hourly captures of ground‐dwelling invertebrates, the ICTs with small shooting areas might underestimate highly mobile invertebrates, such as Araneae, Coleoptera, and Orthoptera, whereas ICTs with large shooting areas might overestimate invertebrates with poor mobility, such as Gastropoda (snail). In contrast, ICTs with small shooting areas might fail to capture images of invertebrates that move faster than their shooting speed. For example, one cricket, five Isopods, and some Collembola and oribatid were captured by pitfall traps with saturated salt solution but were not photographed by ICTs in a follow‐up experiment during winter at the end of 2023 (unpublished data). The absence of these taxa suggested that, apart from the shooting areas, there may be other factors that influence the taxonomic richness and abundance of ground‐dwelling invertebrates photographed by ICTs, such as their movement abilities, habitat preferences, and the resolution of pictures. Additionally, to deal with the redundant and repetitive photography of the same vertebrates using ICTs, researchers usually define an individual's effective detection as the same species from one camera within 30 min or consecutive photos of different species (Chen et al., 2019; Li et al., 2021). Using ICTs as a quantitative sampling method seems reasonable for shooting ground‐dwelling invertebrates with good and low mobility in farmlands (Schirmel, Buchholz, et al. (2010)). Given that ICTs have only recently been used for shooting ground‐dwelling invertebrates, the definition of the effective detection of an individual requires further studies.

### 4.3 Recommendation for ICT application in monitoring ground‐dwelling invertebrates

Although the global use of camera traps in wildlife research is growing exponentially (Meek et al., 2015), the utilization and evolution of camera traps in monitoring and collecting data on ground‐dwelling invertebrates have not been documented. The present study was carried out in a vineyard farmland with a relatively homogeneous environmental condition to preliminarily explore the use of ICTs in capturing ground‐dwelling invertebrates. The authors expected that the findings of the present study could provide useful references and suggestions for researchers for ICT application in related studies.

The findings of the present study demonstrate that ICTs are highly recommended and effective for monitoring taxonomic richness, abundance, and density of ground‐dwelling invertebrates across different shooting areas in farmland. The approach offers a non‐invasive, non‐lethal, user‐friendly, and automatic monitoring tool that does not significantly alter habitats such as a pitfall trap, requires frequent visits, or kills lots of small vertebrates. Additionally, an experiment was conducted using pitfall traps with saturated salt solution to collect ground‐dwelling invertebrates in the same vineyard farmland in 2022. However, the pitfall traps captured some froglets along with the invertebrates. The number of froglets captured by the pitfall traps was even higher than the number photographed by ICTs (unpublished data). The frogs are key predators that contribute to pest control. In fact, removing or killing frogs using pitfall traps in farmland might have more serious consequences than anticipated. Therefore, the contribution of ICTs to biodiversity conservation might be more significant than previously thought, particularly during frog breeding seasons. Furthermore, ICTs have enabled the detection of other vertebrates, such as birds, snakes, mice, and hedgehogs (Figures S1 and S2), which are often overlooked when researchers investigate ground‐dwelling invertebrates using traditional and classical methods. Therefore, the use of ICTs provides an opportunity for the study of the relationships between vertebrates and ground‐dwelling invertebrates in farmland, as well as the top‐down control mechanisms of predators on biodiversity maintenance for ground‐dwelling invertebrates.

Compared to pitfall traps, hand sorting, sweeping, suction samplers, and other classical methods that are typically used to collect ground‐dwelling invertebrates, ICTs theoretically monitor numerous recapture events for invertebrates. However, determining the invertebrates that were recaptured by ICTs is challenging owing to their small body size and the relatively low picture resolution of current technologies. In the present study, a 5‐min interval was used to monitor the ground‐dwelling invertebrates. Based on the experiences when identifying and accounting for the pictures, a 5‐min interval proved relatively suitable for recording Formicidae, Araneae, Coleoptera, Chilopoda, Diplopoda, and Orthoptera because the invertebrates rarely appeared in the photographs taken after a 5‐min delay (or interval). Similarly, a 5‐min interval was used to observe wolf spiders (Lycosidae: *Lycos*a spp.) in the Simpson Desert of central Australia (Potter et al., 2021), and the setting determined the diel activity patterns and habitat use of the relatively large arthropods successfully. However, determining whether it was a recapture of Oligochaeta, Gastropoda (slug), and Gastropoda (snail) was challenging, as certain individuals of the taxa were still visible in the photos taken after a 5‐min delay (interval). In such a scenario, effort was made to determine whether the invertebrates were indeed the same individuals depicted in the six pictures (three consecutive pictures per trigger × two triggering events) that were photographed at two consecutive triggered events. If they represented the same individual, they were recognized and considered a single invertebrate. Conversely, if they were distinct individuals, they were identified and treated as two separate invertebrates. Therefore, accurately accounting for and identifying invertebrates with limited mobility, as well as managing the likelihood of recapture, posed significant challenges in the application of ICTs for studying ground‐dwelling invertebrates. To address the aforementioned challenges, potential solutions include using camera programming with various time intervals or estimating mean time intervals between triggers (Kühl et al., 2023). For instance, Collett and Fisher (2017) employed different intervals, including 15‐s, 30‐s, 60‐s, and 900‐s intervals. They observed that time‐lapse camera trapping with intervals ranging from 1 to 15 min captured approximately twice as many arthropod taxa per day and a third more individuals per day compared to pitfall traps. Nevertheless, such an approach requires more cameras and often entails a trade‐off between economic cost, power consumption, and monitoring duration. Consequently, carefully designing and evaluating the interval settings when utilizing time‐lapse ICTs to study ground‐dwelling invertebrates is essential.

Capturing consecutive pictures when using ICTs to monitor ground‐dwelling invertebrates is crucial. The number of consecutive pictures determines the number of photos taken per trigger event, which depends on the model of camera traps used (Meek et al., 2012). Some camera traps allow the capture of consecutive pictures when triggered, while others only take one photo at a time. Although a single photo may be sufficient to establish the presence and identity of larger mammals (Meek et al., 2012), according to the results of the present study, a single photo was insufficient for identifying the presence or identity of ground‐dwelling invertebrates. In fact, it was common for the invertebrate to appear in the second or third picture of the three consecutive pictures. In such situation, a single photo might introduce bias in the counting and identification of ground‐dwelling invertebrates, especially for those with higher mobility, such as Formicidae and Araneae. Another key benefit of consecutive pictures is that they facilitate rapid detection of invertebrates for researchers. The invertebrates with small body sizes were relatively small in a picture with soil surface as the background in the present study. It was challenging to detect them in a single photo, especially if the photos were taken at night. In such a case, the moving invertebrates were easier to detect in a picture because their spatial positions changed among the three consecutive pictures. This tip is vital for identifying and counting invertebrates using the human eye and experience. Although setting a camera to capture consecutive pictures can deplete memory and/or batteries rapidly, capturing consecutive pictures whenever a trigger occurs is recommended when using ICTs to investigate ground‐dwelling invertebrates in the field.

The height of the ICTs above the soil surface is a crucial factor when utilizing ICTs to monitor ground‐dwelling invertebrates. The optimal height for positioning ICTs is determined by the target animals, the research objective, and the camera's functionality (Meek et al., 2012). For vertebrates or relatively large animals, it is recommended that the camera height be similar to the average body mass of the animals being targeted to enhance detection rates (Meek et al., 2012). As an example, for small mammals, the standard camera trap height is <50 cm above the ground level (Meek et al., 2012). However, there is no standard camera trap height for monitoring invertebrates using ICTs. Naqvi et al. (2022) established a camera trap height of 46 cm, aiming at a focal flower or small cluster of flowers of the same species. They found the setting effective for monitoring interactions between plants and insects of all sizes. For ground‐dwelling invertebrates, Potter et al. (2021) set a camera trap height of 50 cm above the sand surface to quantify nocturnal activity of desert spiders; Zaller et al. (2015) positioned the camera traps at 67 cm above the soil surface to accurately observe arthropod activity in grasslands; Collett and Fisher (2017) positioned camera traps at 25 cm above the ground to estimate activity of leaf litter arthropods in a forest. In the present study, the ICT heights were set at 45 cm above soil surface using the BG636‐48 M camera. The vertical distance allowed the ICTs to identify invertebrates with body sizes longer than Formicidae in the present study. The results indicated that invertebrates with body sizes smaller than Formicidae: a mean body length of 4 mm may not have been monitored by ICTs in the present study. Some Araneae smaller than Formicidae moved on the soil surface during the experiment; however, they were not detected in the pictures during visual inspection. Therefore, the height of the ICTs should be considered carefully when using them to monitor ground‐dwelling invertebrates. A potential method for determining the suitable camera trap height is to calculate the minimum body size of invertebrates that can be detected in the photographed pictures (Figure S2). However, the process is influenced by camera parameters, body size of the target invertebrates, and camera trap direction; therefore, further studies are required.

Camera trap direction is an essential parameter that must be considered when utilizing ICTs to monitor ground‐dwelling invertebrates. When dealing with mammals, the adjustment of this parameter depends on the sensitivity and the type of ICTs being used. False triggers may occur in the morning as the sun rises and begins to warm sunspots and vegetation, and when the sun shines directly on the ICTs. Consequently, aiming the ICTs toward the south, southeast, or southwest can minimize sun facing and reduce the likelihood of false triggers in the southern hemisphere (Meek et al., 2012). For ground‐dwelling invertebrates, time‐lapse ICTs were utilized, and the trigger mode is not sensitive to ambient temperatures in theory. However, there is currently no unified standard for determining the optimal direction of ICTs for monitoring ground‐dwelling invertebrates. Collett and Fisher (2017) set their camera traps vertically above the soil surface to monitor leaf litter arthropods, while Potter et al. (2021) oriented their ICTs at both 45° and 90° to the soil surface to investigate wolf spiders and other invertebrates. In reality, the angled position of ICTs provided a broader field of view, whereas the vertical position increases the likelihood of capturing and identifying ground‐dwelling invertebrates. As the present study aimed to quantitatively investigate ground‐dwelling invertebrates within different shooting areas, the vertical direction of ICTs was used to measure the areas. Therefore, maintaining the camera trap in a steady direction during experimental periods is vital. According to the findings of the present study, if the shooting area was changed when using ICTs in the field, the recording data of ground‐dwelling invertebrates should be affected and altered significantly. Additionally, the camera trap direction will affect the measurement of body size, movement ability, and other functional traits of ground‐dwelling invertebrates within quantitative shooting areas (Figure S2), which are photographed in pictures. A vertical direction of ICTs and a quantitatively constant shooting area will make the measurement of body size and movement abilities of invertebrates more accurate and easier. Additionally, we suggest leaving open areas under ICTs when using them to investigate ground‐dwelling invertebrates in the future, because the small area around circles resulting from soil particles sagging might affect the activities of some invertebrates in the present study, such as Gastropoda (snail). Subsequently, it might affect the taxonomic richness and abundance of invertebrates between different shooting areas. However, if one of the objectives of a study is to measure body sizes and movement abilities of invertebrates, it should be better to keep the camera trap direction and a vertical direction of ICTs first.

Most studies utilizing ICTs to monitor vertebrates have emphasized detection zone and field of view (Meek et al., 2012), yet paid less attention to quantitative shooting areas. In fact, focusing on the shooting area of ICTs can provide more quantitative information for monitoring ground‐dwelling invertebrates compared to monitoring vertebrates or other invertebrates in flowers. Because ground‐dwelling invertebrates are typically active on a relatively flat surface, it theoretically offers us an opportunity to measure their quantitative traits in the field. For instance, if we obtain the origin of the coordinate system, plane Cartesian coordinates, and the vertical distance from the lens of an ICT to the soil surface, theoretically, we could measure the body length, body width, and the ratio of body width to body length of each invertebrate visible in the photographs (Figure S2). Furthermore, other factors, such as the distance between two invertebrates, difference in body length between two invertebrates, or relative spatial positions of different invertebrates, could also be measured, which would provide valuable information for studying their ecological processes (Meek et al., 2015). Therefore, the smoother the soil surface, the more precise the measurement of body size will be. However, the soil surface is not typically as smooth as it seems and is absolutely heterogeneous, particularly for small invertebrates with corresponding body sizes. Therefore, measuring functional traits of invertebrates from photographs taken by ICTs during field monitoring is still challenging. The application of advanced AI technologies could effectively address such challenges, as demonstrated in several related studies (Tresson et al., 2022).

In the present study, as expected, a larger number of ground‐dwelling invertebrates were obtained by increasing the shooting area. The more intriguing question is what the minimum shooting area should be when using ICTs to ensure accurate data and results. A nested model was more effective for assessing the impact of shooting areas on data collected using ICTs. Unfortunately, we overlooked this aspect in the current study, and further research is warranted. Moreover, there is a balance to be struck among shooting areas, the quality of the observed data, and the resolution of the images. Based on the results regarding the total number of ground‐dwelling invertebrates and their daily and hourly temporal dynamics, we discovered that the optimal shooting area when fixing the vertical height of ICTs to 40 cm was 20 × 20 cm. This area proved beneficial for capturing larger invertebrates, particularly those larger than Formicidae, and allowed for the collection of more comprehensive data on their functional traits.

Using ICTs can yield vast amounts of data for studying various aspects of ground‐dwelling invertebrates, including population ecology, behavioral ecology, conservation biology, and biodiversity maintenance mechanisms in farmland. While ICTs could illuminate the intricate temporal dynamics of invertebrates at daily, hourly, and even minute levels in the present study, the monitoring period was shorter compared to that of pitfall trapping during a specific experimental phase. For instance, it took 24 h for a pitfall trap to capture ground‐dwelling invertebrates over the course of a single day, whereas an ICT was able to photograph them in just 0.24 h in the present study. In theory, the shorter the interval, the longer the monitoring duration using ICTs. Consequently, there exist trade‐offs among long monitoring durations, short intervals, electric power usage, and sufficient storage capacity when employing ICTs to monitor ground‐dwelling invertebrates in the field. Therefore, we propose combining ICTs with traditional methods, such as pitfall traps.

The ICTs did not capture a large number of invertebrates, particularly Formicidae, within the same image. This result was inconsistent with our initial expectations during the experimental design phase. Initially, we took precautionary measures to steer clear of Formicidae nests while positioning the ICTs in each designated area. Furthermore, human interventions, encompassing application of pesticides and herbicides, manual weeding, and grape harvesting, could have disturbed Formicidae nests or posed a threat to invertebrates residing within the vineyard farmland. Consequently, we rarely encountered a multitude of invertebrates within a single image during the counting process. Typically, there was one Formicidae per image, occasionally two, and seldom three or more. We hypothesize that the phenomenon of observing a substantial number of invertebrates within the same picture might be more prevalent in habitats like forests, grasslands, or wetlands, where human disturbance is minimal and the population of ground‐dwelling invertebrates is more abundant. Additionally, researchers have endeavored to develop smart camera traps that are capable of monitoring ectothermic animals (Corva et al., 2023). Therefore, we reiterate our suggestion to utilize AI for the counting and identification of ground‐dwelling invertebrates in relevant research efforts.

ICT security has a crucial impact on the security and integrity of recorded data, leading to potential economic and scientific losses. Theft and vandalism are significant limiting factors when it comes to the global use of ICTs for vertebrate monitoring (Kays & Slauson, 2008). Researchers have proposed various security options to ensure ICT security (Meek et al., 2012). However, in the present study, theft and vandalism were not the primary factors affecting ICT security, as the ICTs installed in the vineyard farmland were well protected by managers and farmers. Instead, the main threat factor was severe weather, specifically strong winds and storms due to typhoons. As a result of such strong winds and storms, four ICTs were blown sideways, and two ICTs stopped recording during different periods. Consequently, the height and orientation of the ICTs were altered, leading to significant changes in shooting areas. Therefore, the affected data could not be used to evaluate the effects of ICT shooting areas on invertebrates. Consequently, when using ICTs to quantitatively monitor taxonomic richness, abundance, density, and functional traits of ground‐dwelling invertebrates, there is a need for secure ICT firmly.

In addition, six researchers spent over 3 months counting and identifying invertebrates from photographed pictures. While most researchers were occupied with classes, acquiring knowledge, and conducting other experiments, the process of counting and identifying was still time‐consuming. We believe that utilizing AI technologies would be an effective approach of addressing the above challenge. Standardized annual camera trapping protocol to monitor ground‐dwelling mammals and relatively larger vertebrates have been formed (Jansen et al., 2014; Meek et al., 2012), and some studies have followed these standards (Gorczynski et al., 2021). At the same time, several studies have evaluated different factors that influence the capturing efficiencies of pitfall traps for ground‐dwelling invertebrates, such as diameter (Brennan et al., 2005), depth (Jiménez‐Carmona et al., 2020; Pendola & New, 2007), color (Buchholz et al., 2010), presence and type of preservative (Luff, 1968), sampling duration (Jung et al., 2019), and sampling intensity (Rivera & Favila, 2022). This study only evaluated the effects of one important factor (i.e., shooting area of ICTs) on the diversity of ground‐dwelling invertebrates. To be developed as a widely used method, more factors that may affect the results of ICT studies should be considered and evaluated, such as the spatial distribution of ICTs (Meek et al., 2012), camera alignment (Swearingen et al., 2023), the number of ICT repetitions, weather recording, other environmental factor recording, data management and analysis, and data storage, also need to be considered and further studied. Ultimately, a standard or protocol for using ICTs to monitor ground‐dwelling invertebrates needs to be established through the efforts of interested researchers.

## 5 CONCLUSIONS

In this study, the effects of shooting area of ICTs on the recorded taxonomic richness and abundance of ground‐dwelling invertebrates in a farm were investigated. The results demonstrated that, compared to ICTs with small shooting areas, ICTs with large shooting areas recorded a significantly higher taxonomic richness and abundance of total ground‐dwelling invertebrates and a higher abundance of the dominant taxon throughout the experimental period. Additionally, compared to ICTs with small shooting areas, those with large shooting areas observed higher daily and hourly captures in the taxonomic richness and abundance of total ground‐dwelling invertebrates and abundance of most taxa. In summary, the results of this study suggest that the shooting area of ICTs affects the recorded taxonomic richness and abundance of ground‐dwelling invertebrates. Further studies should evaluate the effects of other potential factors on the monitoring efficiency of ICTs, such as shooting intervals, height of ICTs, orientation of ICTs, spatial patterns of ICT setup, movement abilities of invertebrates, and ICT security. Based on our results, we suggest that shooting areas of ICTs should be a strong consideration during survey design when they are used to investigate ground‐dwelling invertebrates. Overall, this study provides further information on a useful, novel, and automatic tool to monitor ground‐dwelling invertebrates in farmlands and large, high‐quality datasets for decision‐making to protect biodiversity.

## AUTHOR CONTRIBUTIONS

**Meixiang Gao:** Conceptualization (lead); data curation (equal); formal analysis (equal); funding acquisition (lead); investigation (equal); methodology (equal); project administration (equal); supervision (equal); writing – original draft (lead); writing – review and editing (lead). **Jiahuan Sun:** Data curation (equal); formal analysis (equal); investigation (equal); methodology (equal); writing – original draft (equal); writing – review and editing (equal). **Yige Jiang:** Data curation (equal); formal analysis (equal); investigation (supporting); writing – original draft (equal); writing – review and editing (equal). **Ye Zheng:** Formal analysis (equal); methodology (equal); writing – original draft (supporting); writing – review and editing (supporting). **Tingyu Lu:** Formal analysis (equal); methodology (equal); writing – original draft (supporting); writing – review and editing (supporting). **Jinwen Liu:** Conceptualization (equal); funding acquisition (equal); project administration (equal); supervision (equal); writing – original draft (equal); writing – review and editing (equal).

## CONFLICT OF INTEREST STATEMENT

The authors declare that they have no known competing financial interests or personal relationships that could have appeared to influence the work reported in this paper.

## Supporting information


**Figures S1–S2**.

## Data Availability

The data that support the findings of this study are openly available in Dryad at: https://doi.org/10.5061/dryad.79cnp5j3k.
