# Supplementary material for: Shooting area of infrared camera traps affects recorded taxonomic richness and abundance of ground‐dwelling invertebrates
Source: Ecol Evol. 2024 Apr 30;14(5):e11357. doi: 10.1002/ece3.11357 (PMC11061542; doi:10.1002/ece3.11357)
Supplement: Supplementary file 1 — Figures S1–S2. [file ECE3-14-e11357-s001.docx]

Supporting Information

Title: Shooting area of infrared camera traps affects recorded taxonomic richness and abundance of ground-dwelling invertebrates

Meixiang Gao^a,b^, Jiahuan Sun^a,b^, Yige Jiang^a,c^, Ye Zheng^d^, Tingyu Lu^e^, Jinwen Liu^f*^

^a^ Department of Geography and Spatial Information Techniques, Ningbo University, Ningbo 315211, China

^b^ Donghai Academy, Ningbo University, Ningbo 315211, China

^c^ Shenyang University of Chemical Technology, Shenyang, 110142, China

^d^ Faculty of Electrical Engineering and Computer Science, Ningbo University, Ningbo 315211, China

^e^ College of Geography and Environmental Science, Hainan Normal University, Haikou 571158, China

^f^ Institute of Plant Protection, Jilin Academy of Agricultural Sciences, Changchun 130033, China

**Figure captions**

**Fig. S1** Pictures of some vertebrates and invertebrates photographed by ICTs. (a), (b), (c), (d), (e), (f), (g), and (h) are pigeon, mouse, frog, snake, Oligochaeta, Gastropoda (snail), Chilopoda, and Araneae.

Fig. S2 Illustration of the use of ICTs to capture images of (a) vertebrates in their natural habitat, (b) invertebrates found within a flower, and (c) invertebrates on the ground. Please note that for illustrative purposes, the two pandas, two moths, and two invertebrates depicted are assumed to be identical in body sizes.


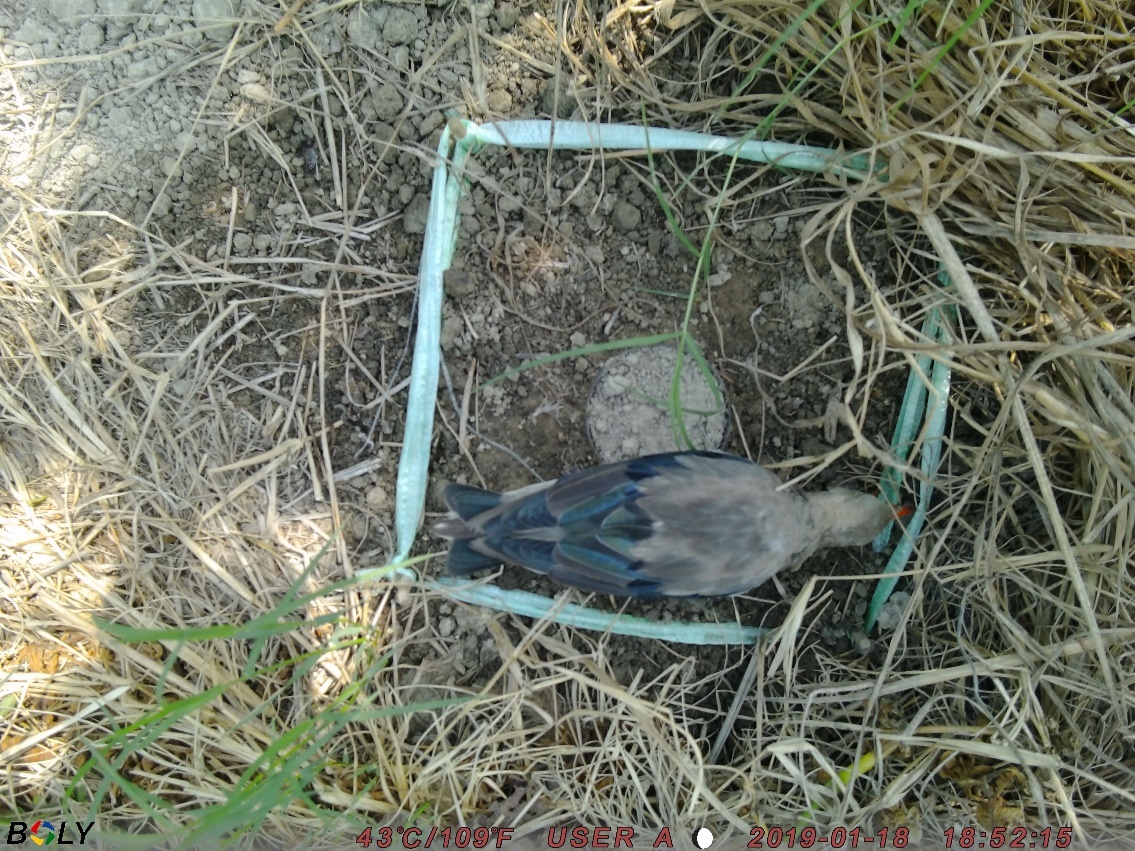
(a)


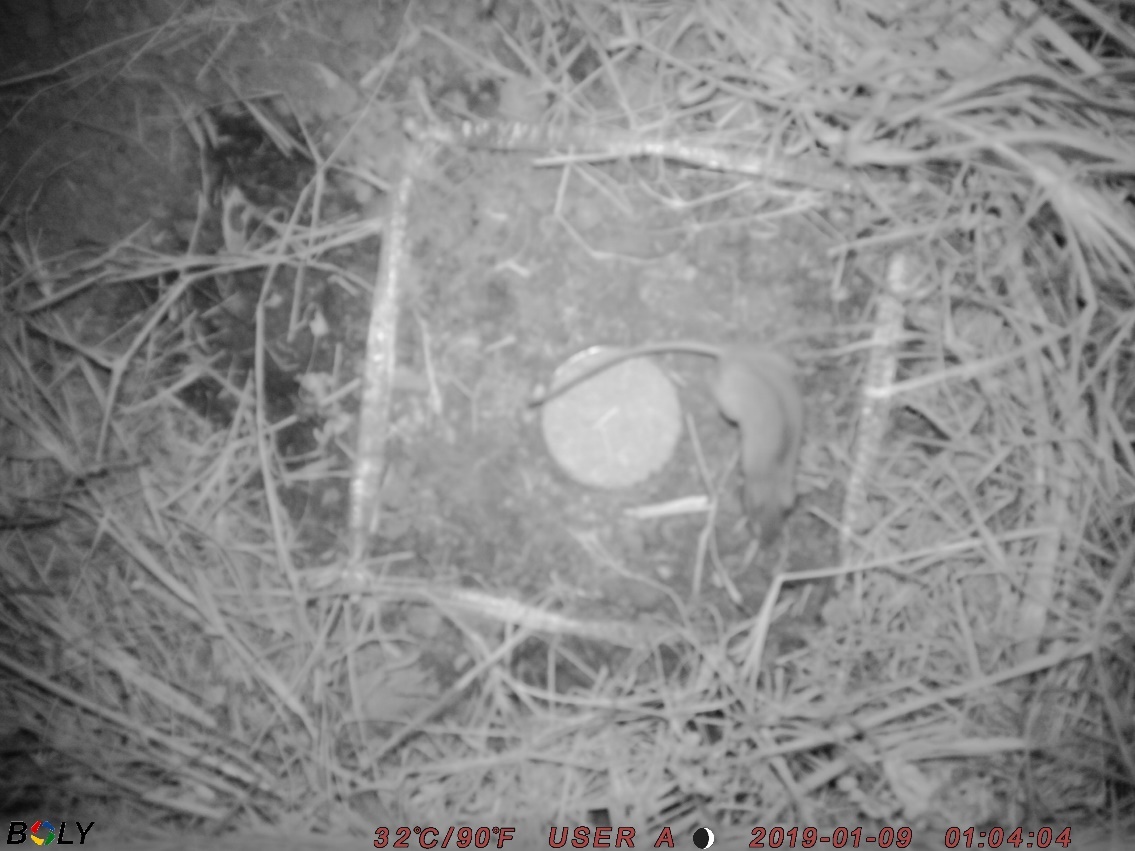
(b)


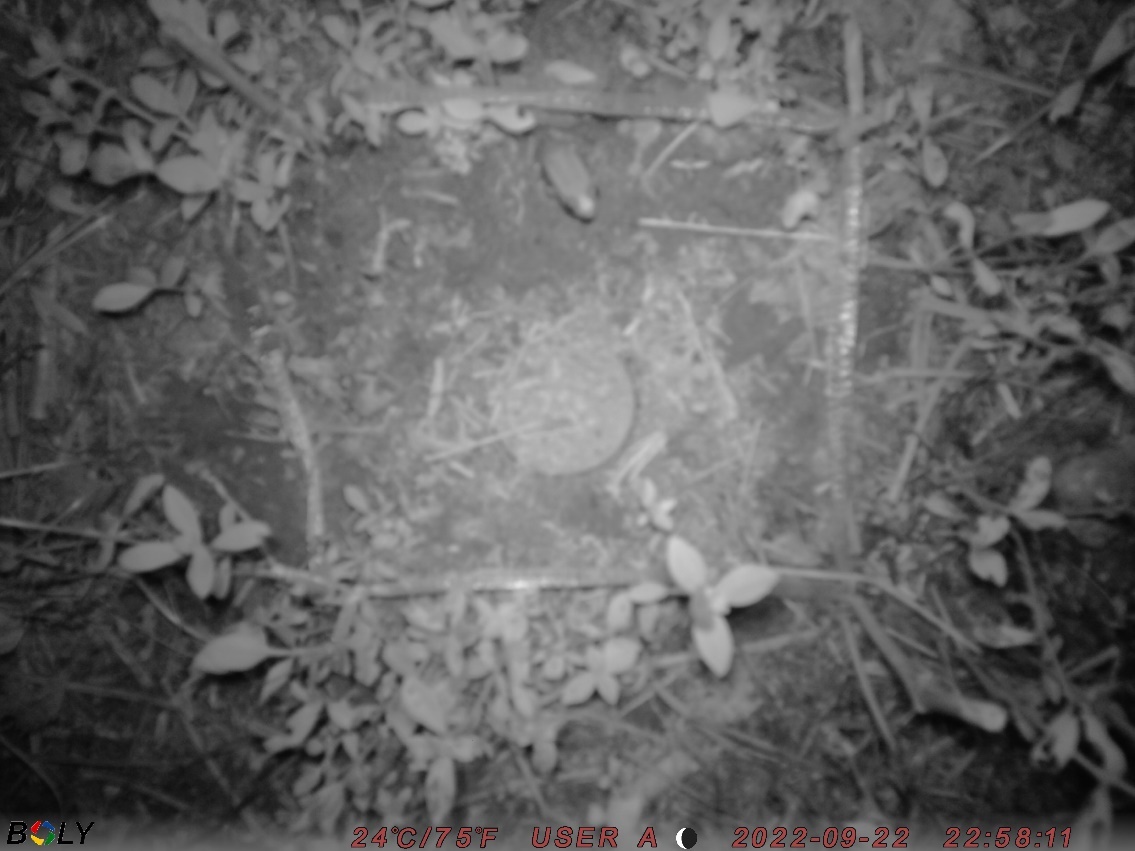
(c)


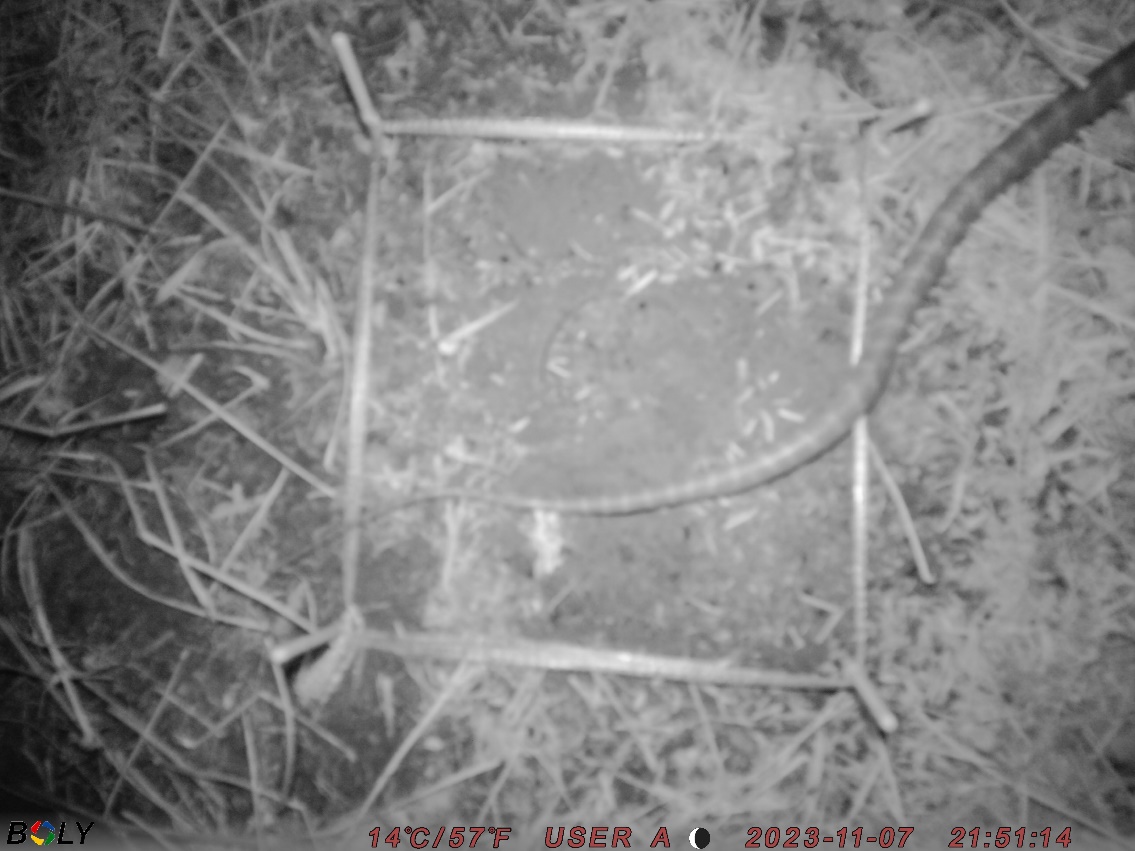
(d)


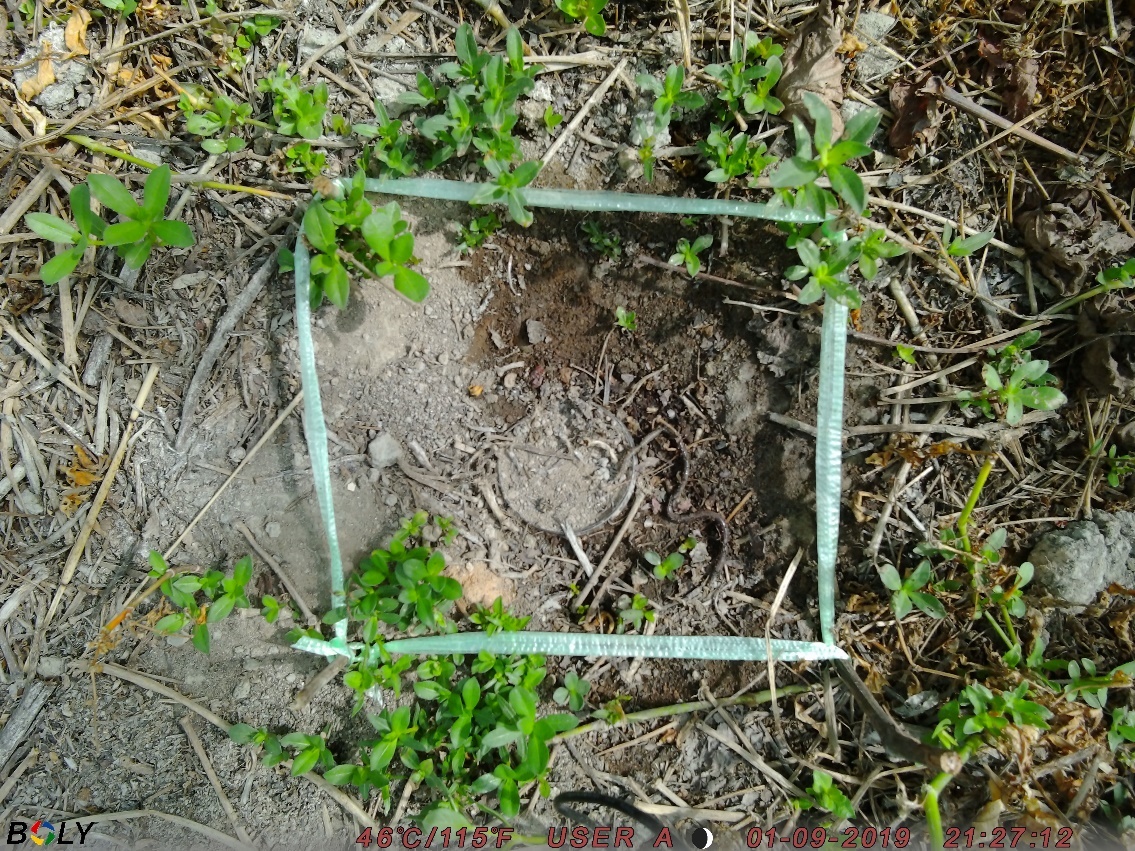
(e)


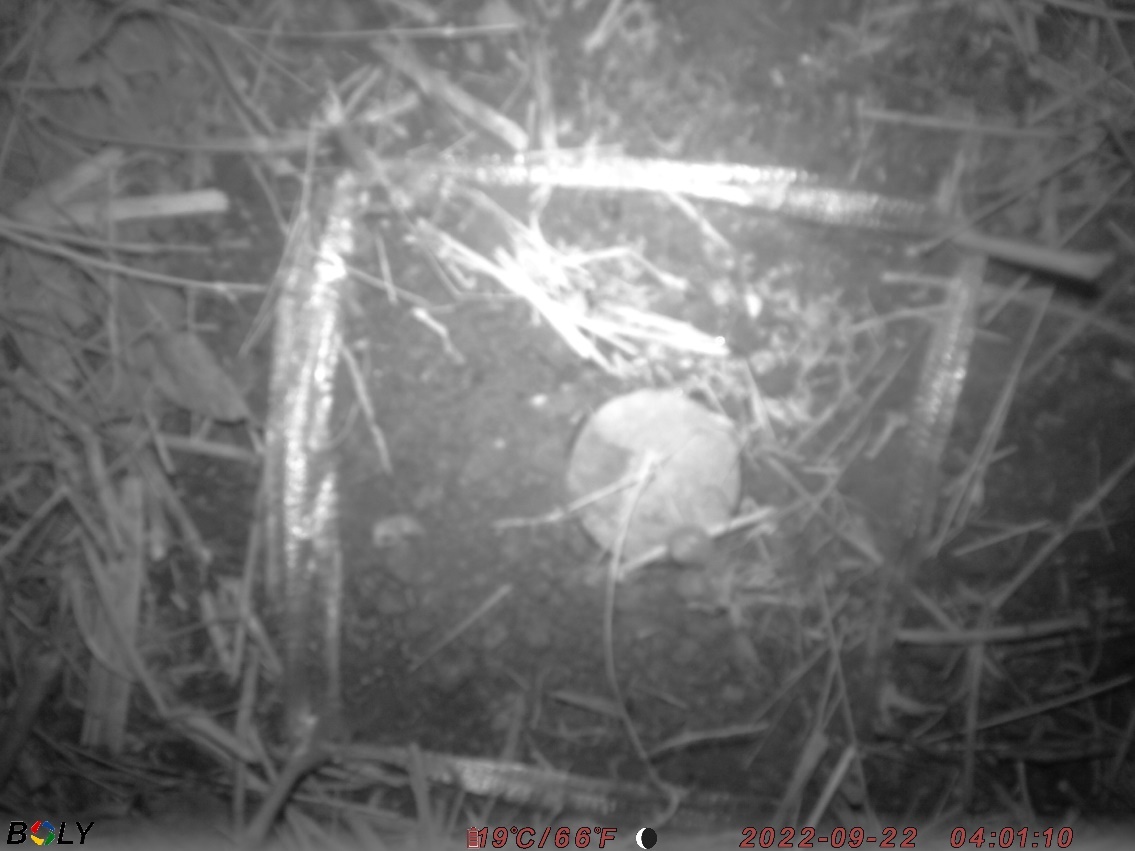
(f)


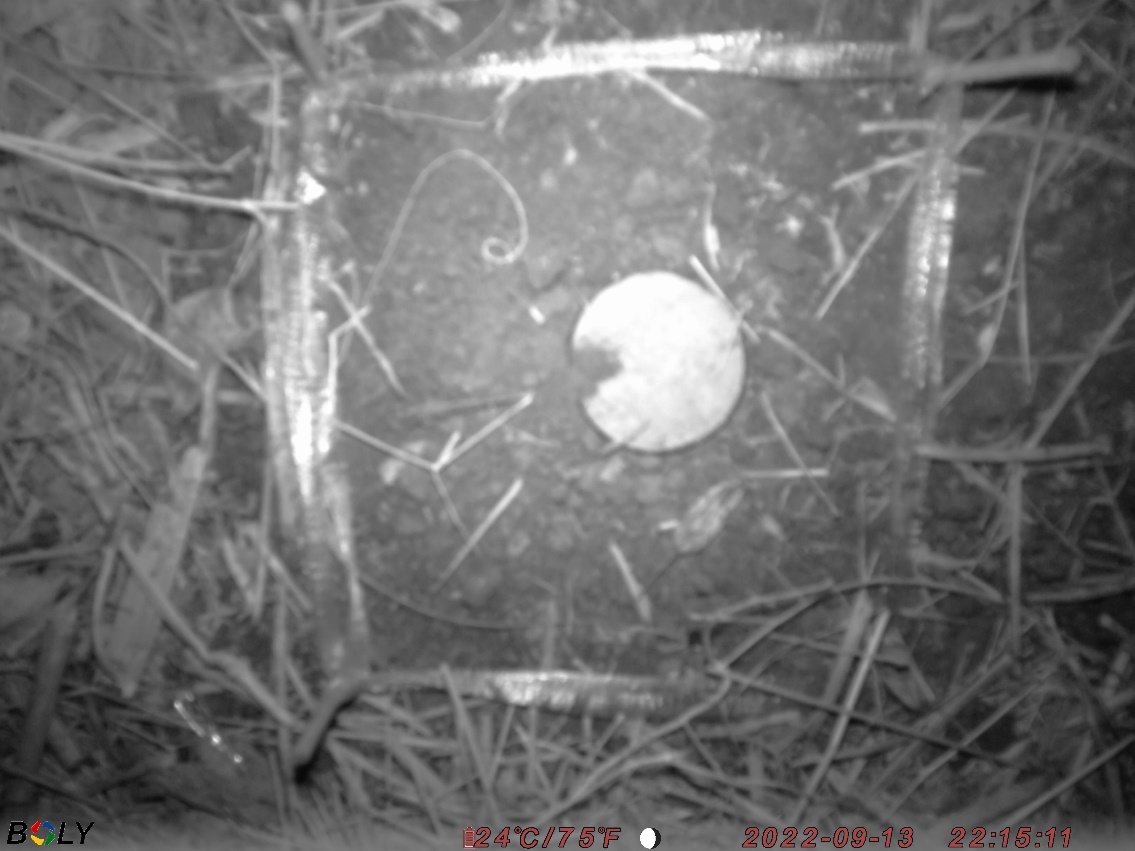
(g)


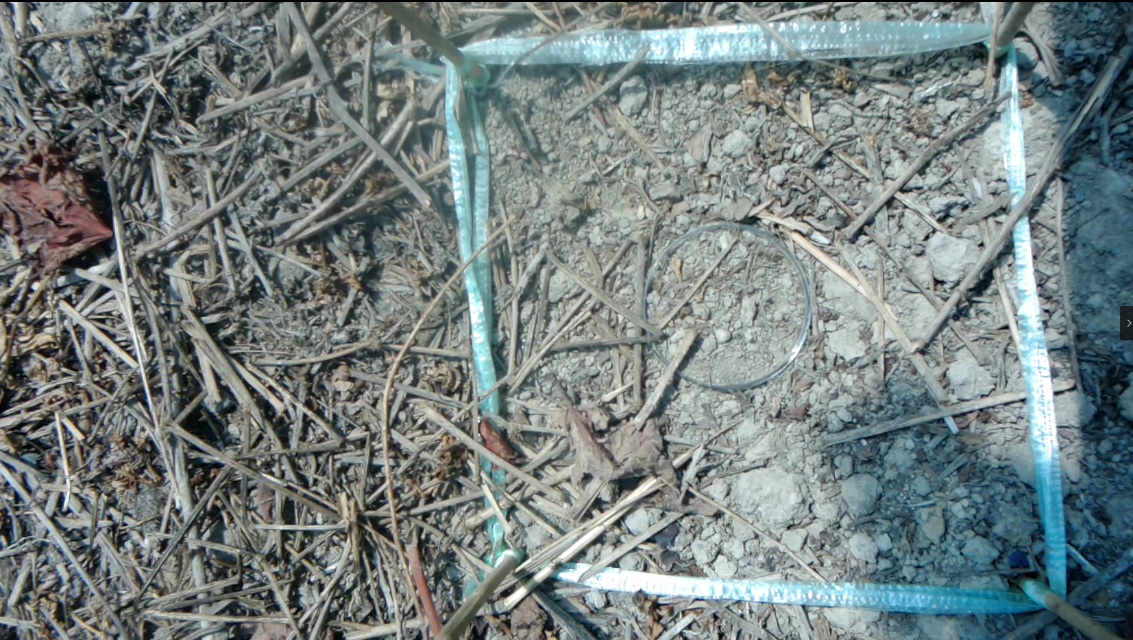
(h)

**Fig. S1** Pictures of some vertebrates and invertebrates photographed by ICTs. (a), (b), (c), (d), (e), (f), (g), and (h) are pigeon, mouse, frog, snake, Oligochaeta, Gastropoda (snail), Chilopoda, and Araneae.


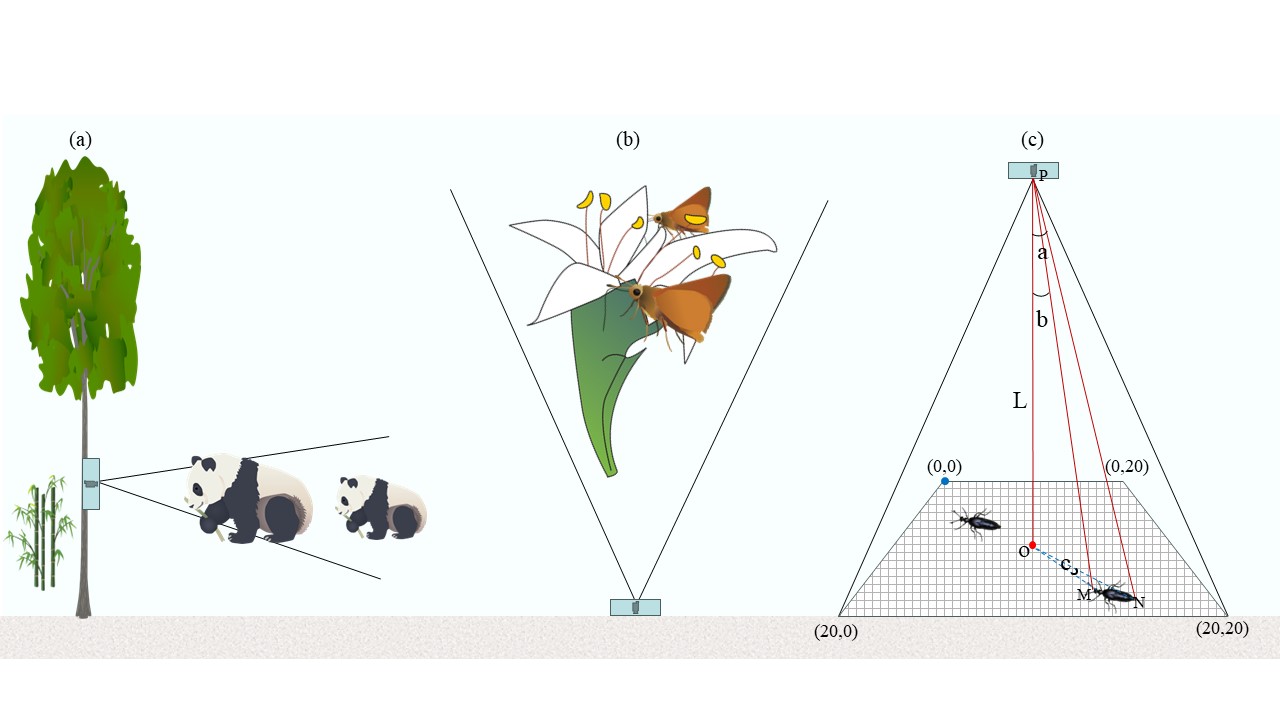


**Fig. S2** Illustration of the use of ICTs to capture images of (a) vertebrates in their natural habitat, (b) invertebrates found within a flower, and (c) invertebrates on the ground. Please note that for illustrative purposes, the two pandas, two moths, and two invertebrates depicted have identical body sizes.
